# State-Dependent Molecular Dynamics

**DOI:** 10.3390/molecules191016122

**Published:** 2014-10-09

**Authors:** Ciann-Dong Yang, Hung-Jen Weng

**Affiliations:** Department of Aeronautics and Astronautics, National Cheng Kung University, Tainan 701, Taiwan; E-Mail: principlex@yahoo.com.tw

**Keywords:** molecular dynamics, state-dependent, quantum Hamilton mechanics, diatomic molecules, Bohmian mechanics

## Abstract

This paper proposes a new mixed quantum mechanics (QM)—molecular mechanics (MM) approach, where MM is replaced by quantum Hamilton mechanics (QHM), which inherits the modeling capability of MM, while preserving the state-dependent nature of QM. QHM, a single mechanics playing the roles of QM and MM simultaneously, will be employed here to derive the three-dimensional quantum dynamics of diatomic molecules. The resulting state-dependent molecular dynamics including vibration, rotation and spin are shown to completely agree with the QM description and well match the experimental vibration-rotation spectrum. QHM can be incorporated into the framework of a mixed quantum-classical Bohmian method to enable a trajectory interpretation of orbital-spin interaction and spin entanglement in molecular dynamics.

## 1. Introduction

One of the greatest challenges in molecular dynamics (MD) is to model processes involving many degrees of freedom, some of which have to be treated quantum mechanically. The combined quantum mechanics (QM) and molecular mechanics (MM) approach to MD [1,2] provides tremendous computational advantages over full quantum mechanical models by treating a limited region of a molecular system quantum mechanically, while treating the rest of the system by using conventional MM methods. The QM part corresponds to what is to be studied in detail, such as the regions involving charge transfer, electron excitations or chemical reactions. The atoms in the QM part are explicitly expressed as electrons and nuclei such that their motions are described quantum mechanically. Quantum effects in MD simulation have been taken into account by various techniques, such as pseudo-spectral methods [3], path integral methods [4], Car–Parrinello-type simulations [5], molecular wave packet dynamics [6] and density matrix evolution method [7]. The performance of a hybrid QM/MM method has been extensively investigated by Billing and coworkers [8]. Brickmann and Schmitt [9] employed a sequence of approximations to transfer the mixed QM/MM dynamics into a Hamilton–Jacobi-type scheme, which is able to formulate the equations of motion by using a single Hamiltonian.

The QM/MM approach to MD simulation of molecular systems mainly relies on the Born–Oppenheimer approximation that a molecular motion can be separated into two independent motions: one is the classical atomic motion on a single adiabatic potential energy surface and the other is the quantum electronic motion in the presence of a time-dependent potential generated by the moving classical atoms. However, there are a huge number of problems for which the interaction between classical and quantum motion is so significant that the Born–Oppenheimer approximation may become invalid. For such problems, the QM/MM approach to MD simulation inevitably faces the crucial issue of self-consistency. The quantum degrees of freedom must evolve correctly under the influence of the surrounding classical motion, and meanwhile, the classical degrees of freedom must respond correctly to the quantum transitions. Therefore, a self-consistent QM/MM approach has to consider not only the effect of the classical degrees of freedom on the quantum ones but also the backreaction of the quantum effect on the classical degrees of freedom.

Two mixed quantum-classical methods that have been developed in the literature to treat the interactions between classical and quantum systems in a self-consistent way are the mean-field method [10] and the surface-hopping method [11]. The mean-field method calculates the force for the classical motion by averaging over the quantum wave function. This method is invariant to the choice of quantum representations and applicable to both bound and continuum states, but it suffers from the neglect of correlations between classical and quantum degree of freedom. On the other hand, the surface-hopping method was developed to manifest quantum-classical correlation, but it is not invariant to the choice of quantum representations and is intrinsically limited to discrete quantum states. Although both methods have their respective limitations, they have been proven to be successful in many applications.

In the latest decade, a novel solution to the quantum backreaction problem in a mixed QM/MM simulation has been proposed using the Bohmian formulation of quantum mechanics [12,13]. The mixed quantum-classical Bohmian (MQCB) approach combines the merits of the above two methods and gives a consistent treatment of mixing quantum and classical degrees of freedom without reference to any basis set. The MQCB method has been applied to the process of vibrational decoherence of I_2_ in a dense helium environment [14], to the case of rotational diffractive surface scattering of a diatomic molecule [15] and to a model of O_2_ interacting with a Pt surface [16], all with good agreement with the full quantum-mechanical treatments.

The current formulation of Bohmian mechanic is mainly based on Cartesian coordinates, which is inconvenient to the description of molecular angular motions. This paper aims to incorporate quantum Hamilton mechanics (QHM) [17,18,19] into the framework of the MQCB method to enhance its capability of handling orbital and spin angular motions. It is well known that the Bohmian velocity vanishes in all of the stationary states with zero angular quantum number, and therefore, the corresponding Bohmian particles will remain standing at their initial positions at any time [20]. QHM, on the one hand, conquers the problem of Bohmian stationarity by formulating Bohmian mechanics in complex space, and on the other, describes MD in terms of Hamilton equations of motion, which are coordinate-independent and suitable to any curvilinear coordinates.

With the proposed modifications by QHM, the computational procedures of MQCB developed in the literature can be used to simulate molecular dynamics, including vibration, rotation and spin motions. The correctness of the derived state-dependent molecular dynamics will be verified by comparing with the quantum mechanical description of a diatomic molecule for which the Schrödinger equation has an analytical solution. Of significance is that the spin dynamics, which cannot be described by spatial wave functions, emerges naturally from the established state-dependent molecular dynamics.

In Section 2, we introduce the working equations of the MQCB method to describe the motion of a diatomic molecule in Cartesian coordinates. QHM is then introduced in Section 3 to reformulate Bohmian mechanics under spherical coordinates. We show that QHM is a single mechanics simultaneously playing the roles of QM and MM by pointing out that QHM comprises two sets of Hamilton equations with the first set describing the vibration-rotation motion of the molecule and the second set describing the time evolution of the wave function. State-dependent vibrational dynamics fully consistent with QM is derived from QHM in Section 4. Section 5 demonstrates how to incorporate molecular spin dynamics into MD simulation under the same motion space governed by QHM. The resulting state-dependent molecular dynamics is found to agree with the prediction of QM and well match the experimental vibration-rotation spectrum.

**Figure 1:**
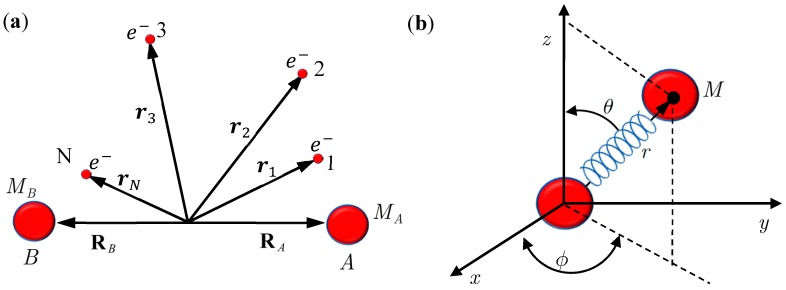
(**a**) A diatomic molecule composed of Nuclei A and B, together with a number of *N* electrons; (**b**) An equivalent single-particle model of the diatomic molecule with rotation and vibration motion described by the spherical coordinates.

## 2. Mixed Quantum-Classical Mechanics

In this section, we review the basics of the MQCB method by applying it to simulate the motion of a diatomic molecule, which is composed of Nuclei A and B, of mass *M_A_* and *M_B_* together with a number *N* of electrons (see Figure 1). The internuclear position vector will be denoted by **X** and the position vectors of the electrons with respect to *O*, the center of mass of A and B, by **r**_1_, **r**_2_,⋯, **r***_N_*. The position vectors of A and B are denoted by **R***_A_* and **R***_B_*, so that = **R***_A_* − **R***_B_*. The Schrödinger equation describing the motion of the molecule takes the following form [12,13]:

(2.1) $$i\hbar \frac{\partial }{\partial t}\Psi (t,x,X)=\left[-\frac{\hbar^{2}}{2m}\frac{\partial^{2}}{\partial x^{2}}-\frac{\hbar^{2}}{2M}\frac{\partial^{2}}{\partial X^{2}}+V(x,X)\right]\Psi (t,x,X)$$

where **x** = [*r*_1_ *r*_2_ ⋯ *r*_3_] is the collective vector representing the quantum motion of the electrons and **X** is the vector representing the classical motion of the nuclei.

Writing the wave function as *Ψ*(*t*,**x**,**X**) = *R*_B_(*t*,**x**,**X**) exp (*iS*_B_(*t*,**x**,**X**)) with *R_B_* and *S_B_* being real, Equation (2.1) can be separated into three coupled equations in terms of *R_B_*, *S_B_* and their derivatives. For a computed *S_B_*, the Bohmian velocity is given by:

(2.2) $$\frac{dx}{dt}=\frac{1}{m}\frac{\partial S_{B}(t,x,X)}{\partial x},\;\frac{dX}{dt}=\frac{1}{M}\frac{\partial S_{B}(t,x,X)}{\partial X}$$

where *m* is the mass of electron and *M* = *M_A_M_B_*/(*M_A_* + *M_B_*) is the reduced mass of the molecule. With the introduction of the quantum potential:

(2.3) $$Q(t,x,X)=-\frac{\hbar^{2}}{2m}\frac{1}{R_{B}}\frac{\partial^{2}R_{B}}{\partial x^{2}}-\frac{\hbar^{2}}{2M}\frac{1}{R_{B}}\frac{\partial^{2}R_{B}}{\partial X^{2}}$$

Equation (2.2) can be recast into classical-like equations of motion:

(2.4) $$\frac{d^{2}x}{dt^{2}}=-\frac{1}{m}\frac{\partial \left(V(t,x,X)+Q(t,x,X)\right)}{dx}$$

(2.5) $$\frac{d^{2}X}{dt^{2}}=-\frac{1}{M}\frac{\partial \left(V(t,x,X)+Q(t,x,X)\right)}{dX}$$

A workable scheme of MQCB has been implemented successfully by replacing the full-dimensional wave function *Ψ*(*t*,**x**,**X)** by a wave function 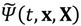 in the **x** quantum subspace that depends parametrically on the classical position **X**(*t*). The approximate wave function 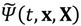 obeys the Schrödinger equation:

(2.6) $$i\hbar \frac{d}{dt}\tilde{\Psi }(t,x,X(t))=\left[-\frac{\hbar^{2}}{2m}\frac{\partial^{2}}{\partial x^{2}}+V(x,X(t))\right]\tilde{\Psi }(t,x,X(t))$$

where *d* / *dt* denotes the material derivative ∂ / ∂*t* + *Ẋ* ∙ 𝛻 along the classical trajectory **X**(*t*). The MQCB method and the mean-field method share the same quantum degree of freedom described by Equation (2.4), but differ from each other in the way that the classical degree of freedom is governed by the quantum wave function. Instead of Equation (2.5), the classical trajectory **X**(*t*) for the mean-field method is computed by the mean potential:

(2.7) $$\frac{d^{2}X}{dt^{2}}=-\frac{1}{M}\frac{\partial }{dX}V_{\text{eff}}(t,X)=-\frac{1}{M}\frac{\partial }{dX}\int {\left|\Psi (t,x,X)\right|}^{2}V(x,X)dx$$

Upon applying Equation (2.5) or Equation (2.7) to a diatomic molecule, we find that it is convenient to express the vector **X** in the spherical coordinates (*r,θ,ϕ*) in order to describe the vibration and rotation of the two atoms with respect to their center of mass. However, the expressions of quantum motion described by Equation (2.2) to Equation (2.7) are valid only for Cartesian coordinates. To give an explicit manifestation of the orbital and spin angular motion of the molecules, the reformulation of Bohmian mechanics under spherical coordinates is necessary.

It will be shown in the next section that QHM provides MQCB with state-dependent molecular dynamics, which preserve the quantization property of orbital and spin angular momentum. By contrast, in the mean-field method, the classical trajectories **X**(*t*) over different quantum states are averaged out to give a state-independent result, as can be seen from Equation (2.7). To compare with the QHM formulation discussed later, here let us have a quick look on the description of the vibration-rotation motion of a diatomic molecule by the classical Equation (2.7). With the effective potential *V*_eff_(*t*,**X**) = *V*_eff_(*t*,*r,θ,ϕ*) given by Equation (2.7), the classical Hamiltonian governing the 3D relative motion of the two nuclei with respect to their center of mass can be expressed by:


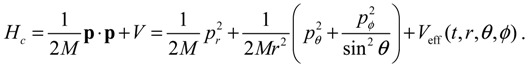
(2.8)

The molecular dynamics (*r*(*t*),*θ*(*τ*),*ϕ*(*t*)) can be solved from the Hamilton equations:

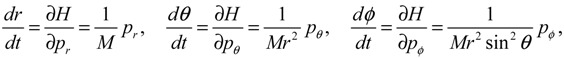
(2.9)

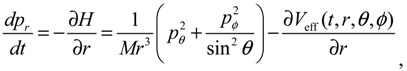
(2.10a)

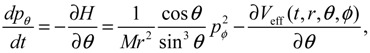
(2.10b)

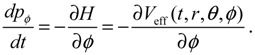
(2.10c)
It is clear that the vibrational and rotational motions of the molecule are determined uniquely by the effective potential *V*_eff_(*t*,*r,θ,ϕ*) with initial conditions (*r*(0),*θ*(0),*ϕ*(0)) and (*p_r_*(0),*p_θ_*(0),*p_ϕ_*(0)). In the case of a central-force potential *V*_eff_(*r*), the total energy *H*_c_, the squared angular momentum *L*^2^ and the z-component angular momentum *L*_z_ are conserved quantities along any dynamic trajectory (*r*(*t*),*θ*(*τ*),*ϕ*(*t*)):

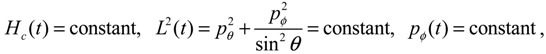
(2.11)
where the three conservation constants depend continuously on the initial conditions. In the next section, a quantum version of the classical Hamilton Equations (2.9) and (2.10) will be derived by QHM. The resulting quantum Hamilton equations can describe molecular dynamics in a general non-stationary quantum state Ψ(*t,r,θ,ϕ*), and a quantum version of the conservation law (2.11) comes out naturally, if Ψ(*t,r,θ,ϕ*) is an eigenfunction.

## 3. Quantum Hamilton Mechanics

Consider the diatomic molecule as shown in Figure 1b. Let **q** be the internuclear position vector expressed in a curvilinear coordinate system, *V*_eff_(*t*,**q**) be the instantaneous internuclear potential, and *M* be the reduced mass. The Schrödinger equation describing the molecular motion can be recast into the quantum Hamilton-Jacobi equation [17,20]:

(3.1) $$\frac{\partial S}{\partial t}+{H_{\Psi }(t,q,p)|}_{p=\nabla S}=\frac{\partial S}{\partial t}+{\left[\frac{1}{2M}p\cdot p+V_{\text{eff}}(t,q)-\frac{\text{i}\hbar }{2M}\nabla \cdot p\right]}_{p=\partial S/\partial q}=0$$

where the action function *S* is the logarithmic wave function defined as:

*S*(*t*,**q**) = −*i*ħln Ψ(*t*,**q**) . (3.2)

The canonical momentum **p** in Equation (3.1) is related to the action function *S* via the law of canonical transformation:

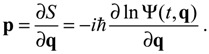
(3.3)
The appearance of the imaginary number 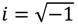 indicates that the momentum **p** has to be defined in a complex domain. In Cartesian coordinates, we have 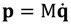 and the particle’s velocity 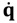 can be determined by the wave function Ψ as

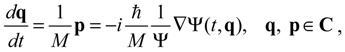
(3.4)
which is the governing equation in the complex-valued Bohmian mechanics [21]. It is the complex momentum **p** from Equation (3.3) rather than the real momentum from Equation (2.2) that matches the momentum distribution provided by standard quantum mechanics [20]. The relationship between the real and complex momentum can be found as:

(3.5) $$\frac{\partial S}{\partial q}=\frac{\partial S_{B}}{\partial q}-i\hbar \frac{1}{R_{B}}\frac{\partial R_{B}}{\partial q}$$

It can be seen that the real Bohmian trajectory solved from 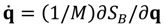 only carry information about the dynamics of the momentum flow, while the complex trajectory solved from 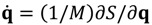 also includes information about the probability *R_B_* = |Ψ|. The dynamics in the complex phase space (**q**,**p**) thus explains in a natural way how to get the correct momentum distribution and explains why algorithms based on complex trajectories are stable and accurate [22,23,24,25,26,27,28]. The advantages of implementing numerical codes in a complex phase space are similar to those of considering complex fields instead of real ones in electromagnetism [20].

When compared with the classical Hamiltonian *H_c_* in Equation (2.8), the quantum Hamiltonian *H*_Ψ_ defined in Equation (3.1) has an additional term called complex quantum potential, which in the state Ψ can be expressed by:

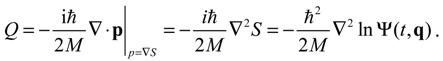
(3.6)
The state-dependent molecular dynamics to be developed here all originate from the state-dependent nature of *Q*. With the quantum Hamiltonian *H*_Ψ_ defined in Equation (3.1), the accompanying Hamilton equations appear as the usual form:

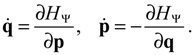
(3.7)
The Hamilton equations are coordinate-independent and valid for arbitrary coordinate systems. Especially, in the Cartesian coordinates, we have ∂*H*_Ψ_/∂**q** = **p**/*M* and the first set of the Hamilton equations (3.7) turns out to be the Bohmian velocity, like the one defined in Equation (2.2).

We now specialize **q** to the spherical coordinates **q** = (*r,θ,ϕ*) with *r* denoting the internuclear distance, and (*θ,ϕ*) denoting the orientation of the molecule. By expanding the inner product **p ∙ p** and the divergence ∇∙ **p** in spherical coordinates, quantum Hamiltonian *H*_Ψ_ in Equation (3.1) can be expressed by [17]:

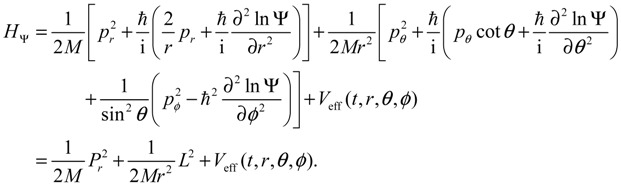
(3.8)
This is the quantum counterpart of the classical Hamiltonian defined in Equation (2.8). Substituting *H*_Ψ_ into Equation (3.7), we obtain the first set of the Hamilton equations as:

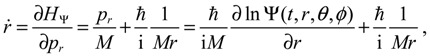
(3.9a)

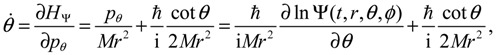
(3.9b)

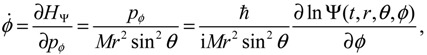
(3.9c)
where the canonical momentum (*p_r_, p_θ_, p_ϕ_*) has been given according to Equation (3.3):

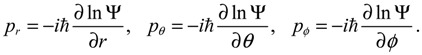
(3.10)
Equation (3.9) is the quantum counterpart of the classical equation (2.9) and provides the vibrational and rotational dynamics (*r*(*t*), *θ*(*τ*), *ϕ*(*t*)) of the molecule in the state Ψ(*t,r,θ,ϕ*), which may be stationary or non-stationary. On the other hand, it can be shown that the second set of Hamilton equation (3.7) with **p** given by Equation (3.3) is just the Schrödinger equation. In other words, the two sets of Hamilton equations (3.7) provide a new approach to MM/QM formulation, where the first set plays the role of MM to derive the molecular dynamics (3.9), while the second set plays the role of QM to give the time evolution of the wave function Ψ.

The quantum Hamiltonian (3.8) can be expressed in a more comprehensive way with the help of Equation (3.9):

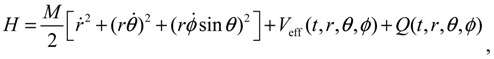
(3.11)
where the first three terms constitute the classical kinetic energy *T*, and the last two terms form the total potential:

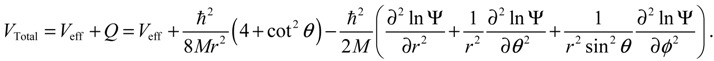
(3.12)
The dynamic equations of motion for the molecule can be described either by the Hamilton equations (3.9) or by the Lagrange equations based on the quantum Lagrangian:

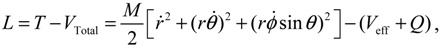
(3.13)
from which the quantum Lagrange equations of motion can be obtained as

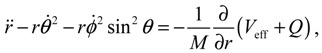
(3.14a)

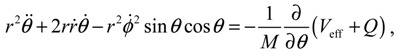
(3.14b)

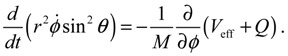
(3.14c)
It can be seen that apart from the internuclear force produced by *V*_eff_, there are additional quantum forces acting on the molecule yielded by the quantum potential *Q*. In the next two sections, we proceed to show that the action of the quantum potential leads to the state-dependent molecular dynamics compatible with the description of the wave function Ψ.

## 4. State-Dependent Molecular Vibration

By replacing Equations (2.2) and (2.5) with Equations (3.9) and (3.14), respectively, the computational procedures of MQCB developed in the literature can be used to simulate molecular dynamics including vibration, rotation and spin motions. Before this new QM/MM approach becomes workable, we have to verify that the governing equations derived in Section 3 yield correct molecular dynamics. The verification is based on the comparison with the quantum mechanical description of a diatomic molecule for which the Schrödinger equation relating to the nuclear motion has an analytical solution. The test model is illustrated in Figure 1a, which is an equivalent single-particle model of a diatomic molecule with rotational and vibrational motion described by the spherical coordinates. The effective internuclear potential is modeled by the Morse function and the corresponding Schrödinger equation is solved analytical in the appendix with eigenfunction Ψ_*n,J,m_J_*_ (*r,θ,ϕ*) = *R_n,J_* (*r*)Θ_*J,m_J_*_ (*θ*)Φ_*m_J_*_ (*ϕ*) given by Equation (A15) and eigenvalue *E_n,J_* given by Equation (A17). All of the parameters and constants appearing below refer to the Appendix.

Substituting Ψ_*n,J,m_J_*_ (*r,θ,ϕ*) into Equation (3.9), we obtain the state-dependent molecular dynamics as:

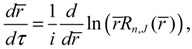
(4.1a)

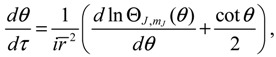
(4.1b)

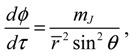
(4.1c)
where *r = βr* is the dimensionless radial distance and *τ = t*(ℏ*β^2^/ M* is the dimensionless time. In a quantum state specified by the three quantum numbers *n*, *J* and *m_J_*, the internuclear distance *r*(*t*) and the molecular orientations *θ*(*τ*) and *ϕ*(*t*) can be expressed as functions of time by solving Equation (4.1).

As a comparison, if the standard Bohmian mechanics based on Equation (2.2) is used to simulate the molecular dynamics in the eigenstate Ψ_*n,J,m_J_*_ (*r,θ,ϕ*), we will find the molecule to be motionless in all the states with *J* = 0. The constitution of the complex momentum in Equation (3.5) explains why it is possible to observe non-vanishing momentum in cases where the Bohmian momentum ∇*S_B_* vanishes.

The first test on the accuracy of the state-dependent molecular dynamics is to examine the quantization laws existing in the eigenstate Ψ_*n,J,m_J_*_ (*r,θ,ϕ*). In the previous section, we have seen that in the conventional MM description, the three conservation constants depend continuously on the initial conditions, being unable to manifest the quantization laws. Now replacing Equations (2.8) and (2.9) by Equations (3.8) and (3.9), we can derive the expected quantization laws. With the eigenfunction Ψ_*n,J,m_J_*_ (*r,θ,ϕ*) given by Equation (A15) and (*p_r_,p_θ_,p_ϕ_*) given by Equation (3.10), the Hamiltonian *H*, the squared angular momentum *L*^2^ and the z-component angular momentum *L_z_* defined in Equation (3.8) can be computed as:

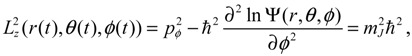
(4.2a)

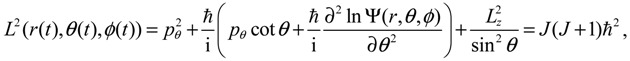
(4.2b)

(4.2c) $$H(r(t),\theta (t),\phi (t))=\frac{1}{2M}\left[p_{r}^{2}+\frac{\hbar }{\text{i}}\left(\frac{2}{r}p_{r}+\frac{\hbar }{\text{i}}\frac{\partial^{2}\ln\Psi (r,\theta ,\phi )}{\partial r^{2}}\right)\right]+\frac{L^{2}}{2Mr^{2}}+V_{\text{eff}}(r)=E_{n,J}$$

We find that the resulting values of 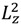, *L*^2^ and *H* are independent of time and are quantized according to the three quantum numbers *n*, *J* and *m_J_*. Unlike the probabilistic interpretation in standard quantum mechanics, here, we have given a dynamic interpretation of the quantization laws by showing that the three physical quantities have constant discrete values along any quantum trajectory (*r*(*t*),*θ*(*τ*),*ϕ*(*t*)) solved from Equation (4.1) in the quantum state specified by the three quantum numbers (*n,J,m_J_*).

The second test is on the consistence between the predictions of the equilibrium bond length made between quantum mechanics and state-dependent molecular dynamics. We will show that the equilibrium bond length *r*_eq_ that maximizes the radial probability *P_r_* satisfies the dynamical equilibrium condition *dr* / *dt* = 0. We first prove this property for the ground vibrational state. Referring to the Appendix, the corresponding radial wave function with *n* = 0 is given by

*R*_0,*J*_ (*r*) = *r*^−1^*e*^−*z*/2^*z*^*α*/2^, *z* = 2*ηe*^−*β*(*r* − *r*_0_)^ . (4.3)

The dependence of *R*_0,*J*_ (*r*) on the angular quantum number *J* is reflected in the relation of *α* and *η* to *J*, as shown in Equation (A14). Substituting *R*_0,*J*_ (*r*) into Equation (4.1a) yields the equation of motion for the rotation-dependent vibration in the ground state:

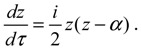
(4.4)
The above radial dynamics has a stable equilibrium point at *z*_eq_ = *α*, *i.e.*,

*r_eq_* = *b* − ln(*α* / 2*η*). (4.5)

The other equilibrium point is at *z*_eq_ = 0, * i.e.*, *r*_eq_ = ∞, corresponding to the condition of molecular dissociation. Equation (4.5) expresses the equilibrium bond length *r*_eq_ as an explicit function of the angular momentum quantum number *J*. This closed-form expression describes analytically how the equilibrium bond length *r*_eq_ increases monotonically with the angular quantum number *J*. Of significance is that the equilibrium bond length *r*_eq_ obtained from the molecular dynamics (4.1a) always coincides with that obtained by QM method. This coincidence has its theoretic origin. We recall the definition of the radial probability:

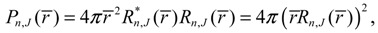
(4.6)
by noting that *R_n,J_* (*r*) given by Equation (A13) is a real function of *r*. On the other hand, according to the dynamic Equation (4.1a), the equilibrium position *r*_eq_ satisfies the condition:

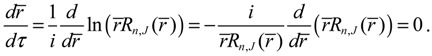
(4.7)
It appears that the equilibrium condition (4.7) is just the condition requiring the radial probability *P_n,J_* in Equation (4.6) to have an extreme value.

All of the properties obtained from the dynamics equation (4.1a) can be re-derived from the action of the radial total force 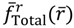. As an illustration, we consider the case with *J* = 0 for which Θ_*J,m_J_*_ (*θ*) = 1 and the total potential defined in Equation (3.12) has a simple expression,

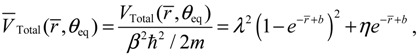
(4.8)
where we note *α* = 2*λ* − 1 and *η* = *λ* from Equation (A14) for the case of *J* = 0. The radial total force 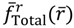 now can be determined from 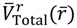 as

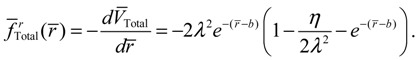
(4.9)
The internuclear distance free from radial force can be found from the condition *f*_*r*_ (*r*) = 0, which leads to

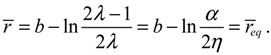
(4.10)
This value is exactly the equilibrium bond length *r*_eq_ already obtained in Equation (4.5) by using the equilibrium condition *dr / dt* = 0.

The evaluation of 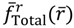 in the vicinity of *r*_eq_ gives 
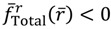
, if *r* > *r*_eq_, and 
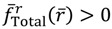
, if *r* < *r*_eq_. The sign of 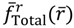 in the neighborhood of *r*_eq_ indicates that the two nuclei attract each other when their distance is longer than *r*_eq_ and repel each other when their distance is shorter than *r*_eq_. Consequently, there is always a restoring force to make *r* return to its equilibrium position *r*_eq_.

**Figure 2:**
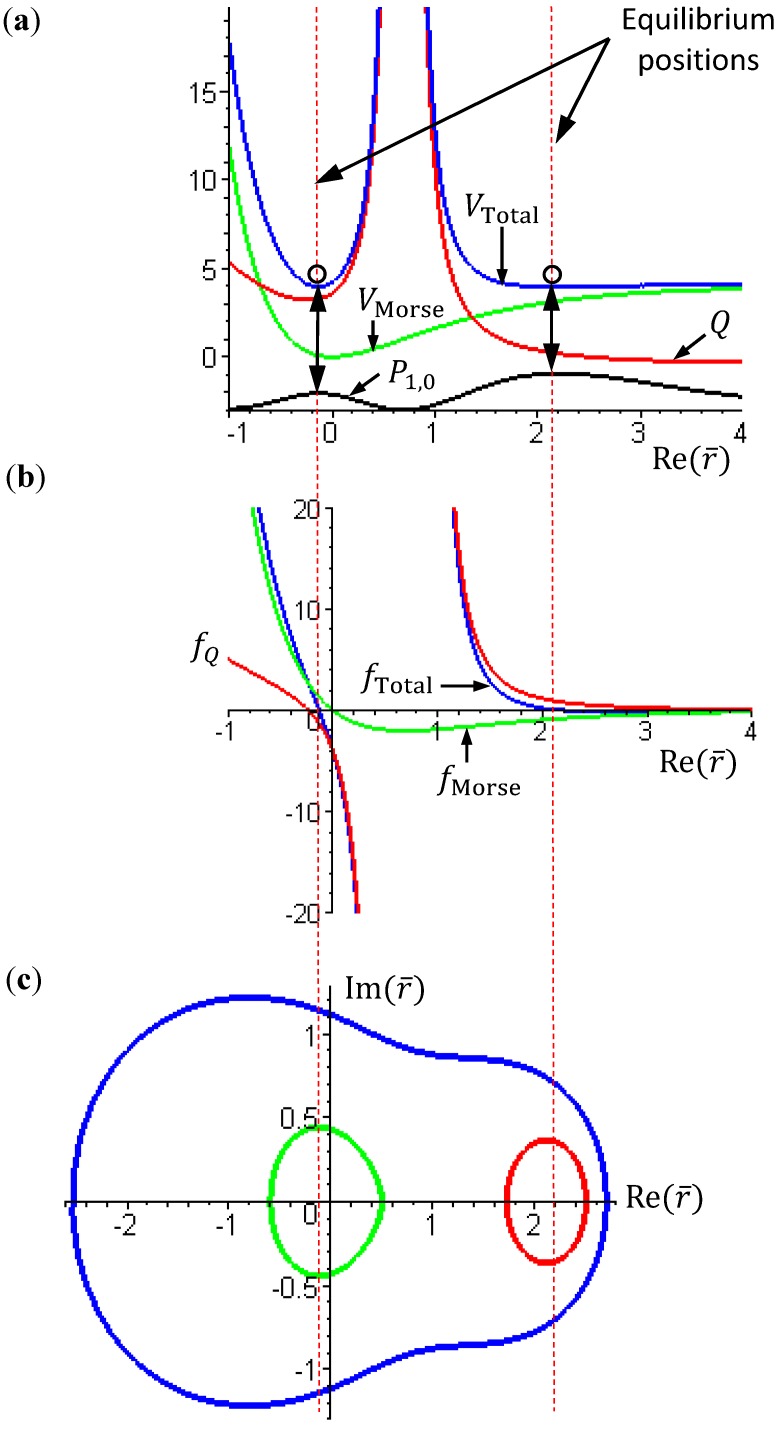
Illustration of the coincidence of four positions for the state with *n* = 1 and *J* = 0: (**a**) The local minimum of the total potential *V*_Total_ and the local maximum of the radial probability *P*_1,0_; (**b**) The position free from the radial force *f*_Total_ (*r*) = 0; (**c**) The equilibrium position with zero velocity *dr* / *dτ* = 0.

Figure 2 gives a numerical verification of the coincidence of four positions for the state with *n* = 1 and *J* = 0: (a) the local minimum of the total potential; (b) the position with *f*_Total_ (*r*) = 0; (c) the equilibrium position with zero velocity *dr* / *dt* = 0; and (d) the local maximum of the radial probability *P*_1,0_, where the first three positions come from the state-dependent molecular dynamics, while the last position from the QM description.

**Figure 3:**
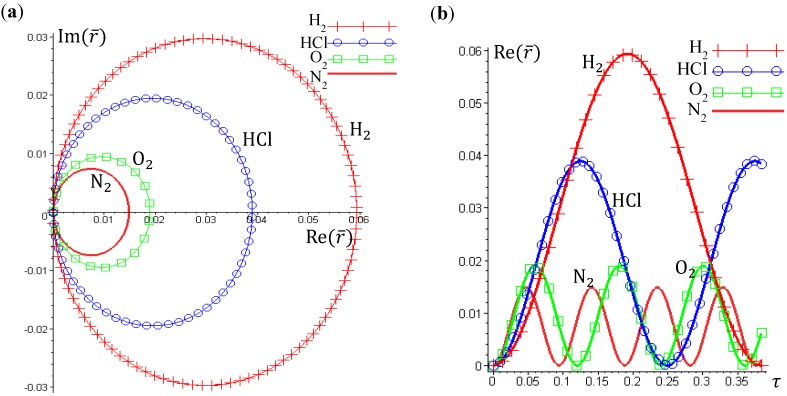
(**a**) The ground-state quantum trajectories solved from Equation (4.4) for the four types of diatomic molecules on the complex plane of *r*. The complex trajectories are closed contours circulating around their respective equilibrium positions *r*_eq_; (**b**) The time responses of Re(*r*(*t*)) give the periods of vibration as *T*_H_2__ = 0.3840, *T*_HCl_ = 1.2496, *T*_O_2__ = 0.1203, and *T*_N_2__ = 0.0941, from which the vibrational frequencies *f* = 1/*T* = ℏ*β*^2^ / (*MT*) are computed and compared with the experimental data listed in Table 1.

The complex trajectories of *r*(*τ*) solved from Equation (4.4) for the four types of diatomic molecules are shown in Figure 3a. It can be seen that the complex trajectories are closed contours circulating about their respective equilibrium positions *r*_eq_ computed from Equation (4.5) by using the molecular parameters listed in Table 1. We find that the period of vibration is independent of the actual trajectories and is quantized with respective to the angular quantum number *J*. This trajectory-independent property can be proven by applying the residue theorem to Equation (4.4):

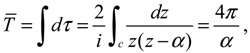
(4.11)
where the contour *c* is an arbitrary closed trajectory solved from Equation (4.4). The contour integral depends only on the poles enclosed by the contours. Because all of the trajectories enclose only one pole at *z* = *α*, the contour integral has only one possible value equal to the residue evaluated at this pole. Due to the dependence of *α* on the angular quantum number *J* as shown in Equation (A14), the resulting period of vibration is allowed only for some discrete values determined by *J*.

Figure 3b illustrates the time responses Re(*r*(*τ*)) for the four diatomic molecules in the ground state *n* = *J* = 0, wherein the periods of vibration have a simple expression

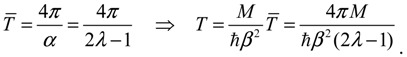
(4.12)
The *λ* values of the four molecules and their periods of vibration computed by Equation (4.12) are listed in Table 1. Also shown in Table 1 are the experimental values *f*_exp_ of the ground-state vibration frequencies. The comparison between the computational and experimental results gives a relative error about 1%, which is caused by the inexistence of an exact solution to the Schrödinger Equation (A1). The radial wave function *R_n,J_* given by Equation (A13) is only a second-order approximation as described by Equation (A6). The approximation gets better as *r* is closer to the equilibrium bond length *r*_0_ of the Morse potential.

**Table 1:** Parameters of four diatomic molecules and the comparison of the computed ground-state vibration frequencies *f*_QHM_ with the measured frequency *f*_exp_.

Diatomic Molecules	H-H	H-Cl	O-O	N-N
Bond length *r*_0_ (m)	74 × 10^−12^	127.5 × 10^−12^	148 × 10^−12^	145 × 10^−12^
Reduced mass (kg/atom)	0.837 × 10^−27^	1.628 × 10^−27^	13.28 × 10^−27^	11.63 × 10^−27^
Potential width *β* (m^−1^)	1.94 × 10^10^	1.81 × 10^10^	2.67 × 10^10^	2.70 × 10^10^
*E*_D_ (kg · m^2^· sec^−2^)	7.11 × 10^−19^	7.39 × 10^−19^	8.28 × 10^−19^	15.77 × 10^−19^
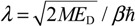	16.8403	25.6722	52.7203	67.2966
*T* = 4*π / (2*λ* − 1)*	0.3845	0.2496	0.1203	0.09406
*T* = *TM* / (ħ*β*^2^)	8.091 × 10^−15^	11.74 × 10^−15^	21.31 × 10^−15^	14.24 × 10^−15^
*f*_QHM_ = 1 / *T*	12,360 × 10^10^	8522 × 10^10^	4693 × 10^10^	7021 × 10^10^
Experiment *f*_exp_	12,470 × 10^10^	8652 × 10^10^	4666 × 10^10^	6987 × 10^10^
|*f*_exp_ − *f*_QHM_| / *f*_exp_	0.8821%	1.503%	0.5786%	0.4866%

The vibration period *T* given by Equation (4.12) can also be derived by using a state-dependent force constant. A quantum force constant is defined as the second-order derivative of the total potential *V*_Total_ (*r*) evaluated at the equilibrium position *r*_eq_:

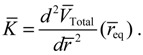
(4.13)
The resulting force constant is state-dependent by noting that *V*_Total_ depends on the three quantum numbers (*n,J,m_J_*) as given by Equation (3.12). A specific *V*_Total_ has been expressed explicitly in Equation (4.8) for the case of *n* = *J* =0, and the substitution of *V*_Total_ into Equation (4.13) yields the force constant as:

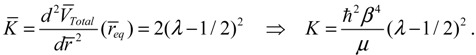
(4.14)
Then the classical relation between the vibration period *T* and the force constant *K* gives:

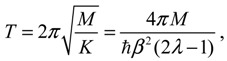
(4.15)
which reproduces the result of Equation (4.12) derived by state-dependent molecular dynamics.

**Figure 4:**
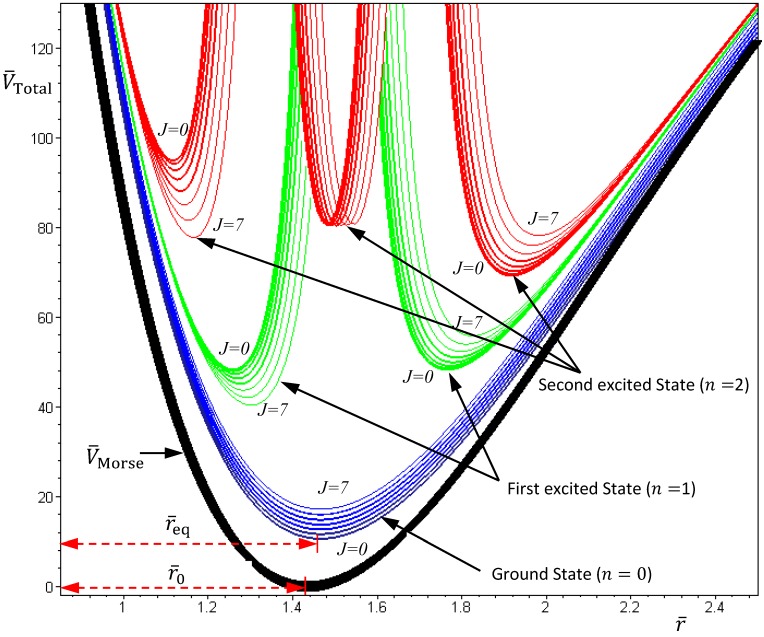
The variation of the total potential *V*_Total_ with respect to the two quantum numbers *n* and *J*. The lowermost points *r*_eq_ of *V*_Total_ correspond to the equilibrium bond lengths, and the curvatures of *V*_Total_ at *r*_eq_ correspond to the force constants of the molecule. This figure schematically illustrates how the bond length and the force constant change due to the change of quantum states.

The above discussions of ground vibrational state reveal a remarkable observation that by replacing the internuclear potential with the total potential *V*_Total_, the conventional MM turns out to be state-dependent MM, which then yields consistent results with QM. This observation applies to every quantum state of the molecule. In every quantum state Ψ_*n,J,m_J_*_, there is a total potential *V*_Total_, which completely determines the molecular quantum dynamics within this state. Figure 4 plots the total potentials for the first three vibrational states and their accompanying rotational states. It can be seen that for a vibrational state with quantum number *n*, there are *n* + 1 sub-shells, within which rotational states with different quantum number *J* reside.

In excited vibrational states *n* ≥ 1, the quantum dynamics (4.1a) has multiple equilibrium points, and the total potential possesses multiple-shell structure. To illustrate this property, we consider the first excited state as an example. The corresponding radial wavefunction is given by:

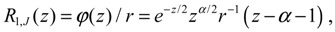
(4.16)
and the radial dynamics is obtained from Equation (4.1a) as:

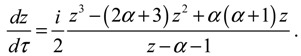
(4.17)
There are two stable equilibrium points in this state, which can be solved from the condition *dz* / *dt* = 0 as:

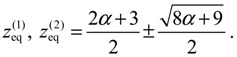
(4.18)
On the other hand, the total potential in the first excited state is given by Equation (3.12):

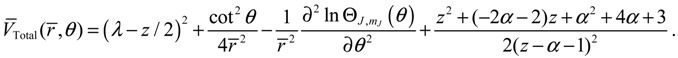
(4.19)
By evaluating the constant *α* at *n* = 1 with *λ* = 2 and *b* = 3, we have:

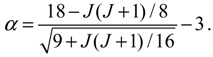
(4.20)
Substituting this *α* into Equation (4.19), the variation of *V*_Total_ (*r*,*θ*_eq_) with respect to the angular quantum number *J* is plotted in Figure 4. It appears that there is a node at *z* = *α* + 1, where the total potential approaches to infinity. This infinite potential barrier separates *V*_Total_ (*r*,*θ*) into two shells. The lowermost point of the inner shell corresponds to the inner equilibrium point 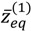, while the lowermost point of the outer shell corresponds to the outer equilibrium point 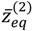, as given by Equation (4.18). Also shown in Figure 4 is the total potential of the second excited state, which has three shells separated by the two nodes.

## 5. State-Dependent Orbital and Spin Dynamics

So far our discussions on molecular quantum dynamics are restricted to the radial vibration motion governed by Equation (4.1a). To take angular dynamics into account, all the three equations in Equation (4.1) have to be considered. In the previous sections we have seen that the QM descriptions of the quantum state Ψ_*n,J,m_J_*_ can be reproduced in terms of the state-dependent molecular dynamics. Here, we go one-step further to show that the spin dynamics, which cannot be described by the spatial wave function Ψ_*n,J,m_J_*_, emerges naturally from the molecular dynamics (4.1) in the ground state where orbital angular momentum is zero.

Setting *n* = *J* = *m_J_* = 0 and substituting *R*_0,0_ (*z*) = *e*^−*z*/2^*z*^*α*/2^/*r*, Θ_0,0_ (*θ*) = Φ_0_ (*ϕ*) = 1 into Equation (4.1) yields the 3D quantum dynamics in the ground state:

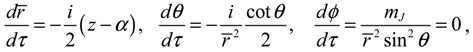
(5.1)
where *α* and *η* are given by Equation (A14) with *J* = 0. We can see that even though the orbital angular quantum number *J* is set to zero, the angular velocity 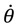 is actually not zero. The main reason why spin motion cannot be attributed to orbital motion in standard quantum mechanics is due to the definition of the orbital angular momentum *L*^2^. To make this point more apparent, we inspect the expression of *L*^2^ defined in Equation (4.2b) with the help of Equation (3.9b):

(5.2) $$L^{2}={\left(Mr^{2}\dot{\theta }\right)}^{2}+\frac{\hbar^{2}}{4}\cot^{2}\theta -\hbar^{2}\frac{\partial^{2}\ln\Psi_{n,J,m_{J}}}{\partial \theta^{2}}+\frac{L_{z}^{2}}{\sin^{2}\theta }=J(J+1)\hbar^{2}$$

In case of *J* = *m_J_* = 0, we have 
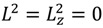
, ∂^2^ lnΨ_*n,J,m_J_*_ (*r,θ,ϕ*) / ∂*θ*^2^ = ∂^2^ ln*R*_*n,J*_ (*r*) / ∂*θ*^2^ = 0 and the above equation is reduced to:

(5.3) $${\left(Mr^{2}\dot{\theta }\right)}^{2}+(\hbar^{2}/4)\cot^{2}\theta =0$$

In the real domain, the only solution is 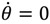 and *θ* = *π* / 2, which is the well-known spinless motion. However, in the complex domain, Equation (5.3) has a nontrivial solution:

(5.4) $$\frac{d\theta }{dt}=-i\frac{\hbar }{Mr^{2}}\frac{\cot\theta }{2}$$

which is just the *θ* dynamics in Equation (5.1) in dimensionless form. In other words, the condition of *L*^2^ = 0 does not completely nullify the complex-valued angular motion, and we can regard the spin dynamics as the remnant angular dynamics as the orbital angular momentum is zero.

**Figure 5:**
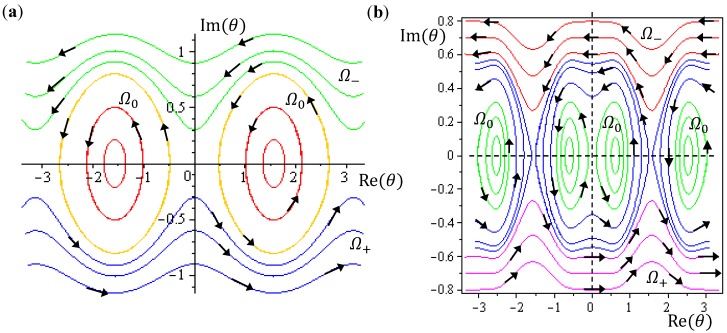
The complex *θ*(*τ*) trajectories for the states (**a**) *J* = 0, and (**b**) *J* = 1 with *m_J_* = 0 solved from Equation (4.1) with *r* at its equilibrium position. The upper region Ω_−_ contains spin-down dynamics with *dθ_R_* / *dτ* < 0, the central region Ω_0_ contains spinless dynamics exhibiting periodic motion around the equilibrium points and the lower region Ω_+_ contains spin-up dynamics with *dθ_R_* / *dτ* > 0.

The integration of the *θ* dynamics with *θ* = *θ_R_* + *iθ_I_* gives the three regions of trajectories as shown in Figure 5a. Within the Ω_0_ region, the sign of *dθ_R_* / *dτ* changes alternatively and produces zero mean velocity. Within the Ω_−_ region, *θ_R_*(*τ*) is monotonically decreasing with a mean value of *dθ_R_* / *dt* equal to −1. Within Ω_+_ region, *θ_R_* (*τ*) is monotonically increasing with a mean value of *dθ_R_* / *dτ* equal to one.

Although orbital angular momentum is completely depressed by the condition *J* = *m_J_* = 0, non-zero angular motion does exist in the regions of Ω_−_ and Ω_+_. To relate this remnant angular motion to spin, the next step is to identify the magnitude of this remnant angular momentum. In the regions of Ω_−_ and Ω_+_, we note the following relations for the complex variable *θ* = *θ_R_* + *iθ_I_*:

cos*θ* = cos*θ_R_*cosh*θ_I_* − *i*sin*θ_R_*sinh*θ_I_* = *e*^|*θ_I_*|^(cos*θ_R_* ∓ *i*sin*θ_R_*) / 2, |*θ_I_*| ≫ 0 , (5.5a)

sin*θ* = sin*θ_R_*cosh*θ_I_* + *i*cos*θ_R_*sinh*θ_I_* = *e*^|*θ_I_*|^(sin*θ_R_* ± *i*cos*θ_R_*) / 2, |*θ_I_*| ≫ 0 . (5.5b)

Therefore, angular momentum in the *θ* direction becomes:

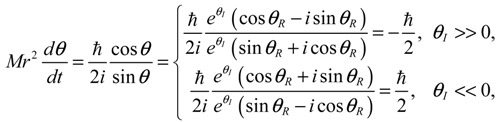
(5.6)
In the region Ω_0_, the *θ*(*τ*) dynamics exhibits periodic motion around the equilibrium points and yields zero mean angular velocity. According to the behavior of the *θ* dynamics, three spin regions can be defined,


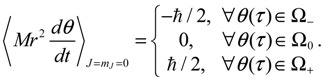
(5.7)

The spin dynamics we discuss so far originates from the wavefunction Ψ = *R_n,J_* (*ρ*)Θ_*J,m_J_*_ (*θ*)Φ_*m_J_*_ (*ϕ*) with Θ_*J,m_J_*_ (*θ*) given by 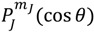, the first-type Legendre function. Indeed, 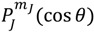 is only a special solution for Θ_*J,m_J_*_ (*θ*) whose general solution can be represented by:

(5.8) $$\Theta_{J,m_{J}}(\theta )=B_{1}P_{J}^{m_{J}}(\cos\theta )+B_{2}Q_{J}^{m_{J}}(\cos\theta )$$

where 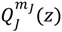 is the second-type Legendre function. It can be shown by integration Equation (4.1) with 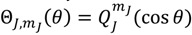 that the direction of the angular motion produced by 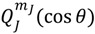 is always anti-parallel to that produced by 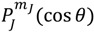. Therefore, in the same quantum state specified by the spatial quantum numbers (*n,J,m_J_*), the molecule can behave either according to the spin dynamics with 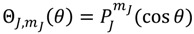 or to the anti-spin dynamics with 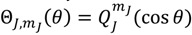. For the former case, we say that the molecule is in a sub-state of (*n,J,m_J_*) denoted by (*n,J,m_J_,1/2*), and for the latter case, the molecule is in the sub-state (*n,J,m_J_,−1/2*). Furthermore, an entangled spin dynamics can be simulated according to the state-dependent molecular dynamics by expressing Θ_*J,m_J_*_(*θ*) as a linear combination of 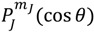 and 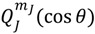 as described by Equation (5.8).

In the ground state, we have seen that the orbital angular motion vanishes and the remnant angular motion in the *θ* direction emerges as the spin dynamics. We proceed to demonstrate that in excited states, orbital and spin motions coexist and both contribute to angular momentum. To examine the interaction between orbital and spin dynamics, we next consider quantum states with *J* = 1 and *m_J_* = 0. Since the *z*-component angular momentum *L_z_* is zero in these quantum states, we are interested in where the orbital angular momentum emerges and how it interacts with the spin angular momentum. The *θ* and *ϕ* dynamics for these quantum states are governed by Equations (4.1b) and (4.1c) as:

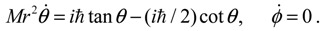
(5.9)
The trajectories of *θ* (*t*) on the complex *θ_R_* − *θ_I_* plane are illustrated in Figure 5b, where it can be seen that as in the ground state, three regions representing three angular motions come about. In the region Ω_−_ (*θ_I_* ≫ 0), *θ_R_* is monotonically decreasing, while in the region Ω_+_ (*θ_I_* ≪ 0), *θ_R_* is monotonically increasing. In the central region Ω_0_, *θ_R_* is a periodic function of time. In conjunction with the relation (5.5), the mean angular momentum of Equation (5.9) becomes:

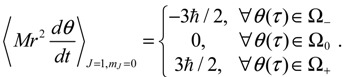
(5.10)
Comparing with Equation (5.7), we find that in the regions of Ω_−_ and Ω_+_, the angular momentum contains an additional component ħ contributed from the orbital motion *J* = 1 apart from the spin angular momentum ħ/2, indicating that the orbital angular motion, in the states with *J* = 1, and *m_J_* = 0, is produced solely by the *θ* dynamics because of 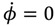.

For a quantum state with quantum number *J* ≠ 0 and *m_J_* ≠ 0, the *θ* and *ϕ* dynamics can be expressed by:

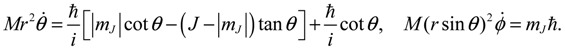
(5.11)
Making use of Equation (5.5) once again, we obtain a general expression for the angular momentum in the *θ* and *ϕ* directions as:

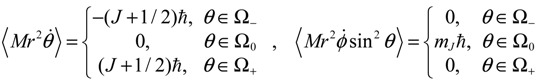
(5.12)
In summary, in the Ω_0_ region, one has zero-mean *θ* dynamics and a quantized *z*-component angular momentum *m_J_*ħ, which is a well-known result in QM; while in the Ω_−_ and Ω_+_ regions, one has an angular momentum ±(*J* + 1/2)ħ in the *θ* direction and a zero-mean orbital angular momentum in the *ϕ* direction, which is otherwise unknown to QM.

The angular momentum given by Equation (5.12), which is derived from the state-dependent molecular dynamics, provides us with a trajectory-based method to determine the rotational energy of a diatomic molecule and its rotational spectrum. The most used formula of rotational energy takes the following form:

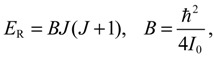
(5.13)
which treats the molecule as a rigid rotor with moment of inertia *I*_0_ = *Mr*_0_^2^ / 2. A typical rotational spectrum consists of a series of peaks corresponding to transitions between adjacent levels satisfying ∆*J* = ±1:

(5.14) $$\nu_{\text{rigid}}=\frac{\Delta E_{R}}{hc}=\frac{B}{hc}(J+1)(J+2)-\frac{B}{hc}J(J+1)=2\bar{B}(J+1),\;\bar{B}=\frac{\hbar^{2}}{\text{4}hcI_{0}}$$

The rigid-rotor model assumes a constant internuclear distance *r*_0_ and neglects the stretch of the bond as the molecule rotates. To account for bond stretching due to rotation, we consider a refined rotation-energy component *E_R_* in the eigenvalue expansion given by Equation (A20), from which the wavenumber of the transition from *J* + 1 to *J* can be derived as:

(5.15) $$\nu_{\text{non-rigid}}=2\bar{B}(J+1)-4\bar{D}{\left(J+1\right)}^{3},\;\bar{D}=\frac{1}{hcE_{D}}{\left(\hbar^{2}/4bI_{0}\right)}^{2}$$

where *D* is a correction factor known as the centrifugal distortion constant. Table 2 lists the measured far infrared absorption spectrum (*ν*_exp_) [29] for the diatomic molecule H-Cl and the predicted spectrum by the rigid model (*ν*_rigid_) and the non-rigid model (*ν*_nonrigid_) with *B* = 10.58 cm^−1^ and *D* = 5.6 × 10^−4^ cm^−1^ determined from the molecular parameters listed in Table 1.

**Table 2:** Comparisons of the four predictions *ν*_rigid_, *ν*_nonrigid_, *ν*_QHM_ and *ν*_fitting_ with the measured rotational spectrum *ν*_exp_.

Transitions	*ν*_rigid_ (cm^-1^)	*ν_non-_*_rigid_ (cm^-1^)	*ν*_QHM_ (cm^-1^)	*ν*_fitting_ (cm^-1^)	*ν*_exp_ (cm^-1^)	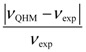
*J* = 0 → 1	21.16	21.16	20.80	20.88	20.88	0.383%
*J* = 1 → 2	42.32	42.30	41.57	41.74	41.74	0.407%
*J* = 2 → 3	63.48	63.42	62.29	62.58	62.58	0.463%
*J* = 3 → 4	84.64	84.49	82.93	83.38	83.32	0.468%
*J* = 4 → 5	105.8	105.52	103.47	104.14	104.13	0.634%
*J* = 5 → 6	126.96	126.48	123.87	124.83	124.73	0.689%
*J* = 6 → 7	148.12	147.35	144.11	145.45	145.37	0.867%
*J* = 7 → 8	169.28	168.13	164.16	165.97	165.89	1.024%
*J* = 8 → 9	186.65	188.81	184.01	186.40	186.23	1.192%

Instead of using the quantum rotational energy obtained from the eigenvalues of Schrödinger equation, we will give an estimate of the wavenumber of transition based on the classical expression of the rotational energy 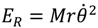 with *r* and 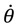 computed by the state-dependent molecular dynamics:

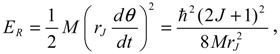
(5.16)
where 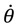 is given by Equation (5.12) and the bond length *r* is denoted by *r_J_* to emphasize its dependence on the angular quantum number *J* as given by Equation (4.5) and Equation (A14). An alternative estimate of the wavenumber caused by rotation absorption transition turns out to be:

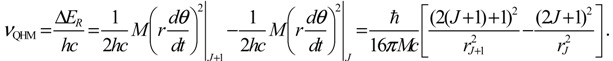
(5.17)
With the molecular parameters of HCl given by Table 1, the bond length *r_J_* is calculated by using Equation (4.5) and then substituted into Equation (5.17) to yield the estimated spectrum *ν*_QHM_.

Table 2 compares the four predictions *ν*_rigid_, *ν*_nonrigid_, *ν*_QHM_ and *ν*_fitting_ with the measured rotational spectrum *ν*_exp_. It can be seen that the wavenumber *ν*_fitting_ is closest to *ν*_exp_, because *ν*_fitting_ is obtained by tuning the parameters *B* and *D*, so that Equation (5.15) best fits the experimental data. The curve fitting gives the best values as B* = 10.44 cm^−1^ and D* = 5.2 × 10^−4^ cm^−1^. The two wavenumbers *ν*_nonrigid_ and *ν*_QHM_ can be compared on the same footing, since both adopt the same molecular parameters listed in Table 1 in the computation. Their difference lies in the model of rotational energy used to compute wavenumbers. The wavenumber *ν*_nonrigid_ is based on the eigen-energy model (A20), while *ν*_QHM_ is based on the QHM model (5.16). Table 2 reveals that the wavenumber *ν*_QHM_ is much closer to the experimental results than *ν*_nonrigid_. 

Theoretically, the description of state-dependent molecular dynamics by QHM is equivalent to QM; however, the actual accuracy depends on the degree of approximation involved in the solution to the wave function. The equilibrium bond length *r_J_* computed from Equation (4.5) is based on the radial wave function *R_n,J_* given by Equation (A13), which is an approximate solution to the radial Schrödinger Equation (A5) by employing the second-order expansion (A6). When the quantum number *J* increases, the bond length deviates further from the equilibrium position *r*_0_ of the Morse potential, and the accuracy of the expansion (A6) gets worse. This tendency explains the degradation of the prediction accuracy of *ν*_QHM_ with respect to the measured rotational spectrum *ν*_exp_.

## 6. Conclusions

A new QM/MM approach called quantum Hamilton mechanics (QHM), is proposed in this paper to establish state-dependent molecular dynamics (SDMD) in such a way that the governing equations of SDMD can be derived by MM with solutions compatible with QM. As a complex extension of Bohmian mechanics, QHM is coordinate-independent and especially suitable in curvilinear coordinates to simulate coupled orbital/spin dynamics. The correctness of SDMD has been verified by comparing with the quantum mechanical description of a diatomic molecule for which Schrödinger equation has an analytical solution. The resulting SDMD simultaneously satisfies the continuous-time dynamics governed by MM and the quantized dynamics governed by QM. QHM can be incorporated into the current framework of the mixing quantum/classical Bohmian (MQCB) method to simulate molecular dynamics. The incorporation of QHM with MQCB enables a trajectory interpretation of orbital-spin interaction and makes it possible for us to simulate spin entanglement in molecular dynamics.

## Appendix

### Solving Schrödinger Equation with 3D Morse Potential

The Schrödinger equation describing the motion of an atomic molecule as shown in Figure 1b takes the following form:

(A1)

Whose solution is expressible in terms of the product:

(A2)

with  denoting the associated Legendre polynomial. The radial function *R*(*r*) satisfies the equation:

(A3)

The effective internuclear potential *E*_eff_(*r*) is modeled by the Morse potential:

*V*_Morse_ (*r*) = *E_D_* [1 − *e*^−*β*(*r* − *r*_0_)^]^2^ ,
(A4)

where *r*_0_ is the equilibrium bond length, *E_D_* is the potential energy for bond formation and *β* is a parameter controlling the width of the potential well. The three parameters *r*_0_, *E_D_*, and *β* are determined from the spectroscopic data.

By rewriting *R*(*r*) = *φ*(*r*) / *r* and changing the independent variable from *r* to *y* = *e*^−*β*(*r* − *r*_0_)^, Equation (A3) can be recast into the following form:

(A5)

This equation has no analytical solution; an approximate approach is to expand *r*_0_^2^ / *r*^2^ with respect to *y* − 1 and retain up to the second-order terms [30]:

(A6)

It is noted that as *y* → 1, we have *r* → *r*_0_. Hence, the accuracy of the approximation (A6) is higher when the molecule is closer to its equilibrium position. With this approximation, Equation (A5) becomes:

(A7)

where *E* = *E* / (*β*^2^ħ^2^ / 2*M*), *E_D_* = *E_D_* / (*β*^2^ħ^2^ / 2*M*), and:

(A8)

In order to transfer Equation (A7) into a standard differential equation, we introduce three new parameters:

(A9)

and express the solution *φ*(*y*) to Equation (A7) in terms of the following form:

*φ*(*z*) = *e*^−*z*/2^*z*^*α*/2^*F*(*z*), *z* = 2*ηy* = 2*ηe*^−*β*(*r* − *r*_0_)^ .
(A10)

Then Equation (A7) can be reduced to the hypergeometric equation:

(A11)

To have a bound solution to the hypergeometric equation, the following condition must be imposed on the constants *k* and *α*:

(A12)

where the integer *n* is known as the vibrational quantum number. Under this condition, the radial wave function turns out to be:

*R_n,J_* (*r*) = *r*^−1^*e*^−*z*/2^*z*^*α*/2^*F*(−*n*,*α* + 1;*z*), *z* = 2*ηe*^−*β*(*r* − *r*_0_)^,
(A13)

where *F*(−*n*,*α* + 1;*z*) is the *n*^th^-order hypergeometric polynomial. The parameters *α* and *η* in *R_n,J_* (*r*) are determined by the two quantum numbers *n* and *J* as:

(A14)

The substitution of the radial wave function *R_n,J_* (*r*) into Equation (A2) gives the full wave function:

(A15)

The bound condition (A12) leads to the quantization of energy. Inserting the definition of *α* from Equation (A9) to Equation (A12), we obtain:

*α*^2^ = (*k* − 2*n_v_* − 1)^2^ = −4(*E* − *E*_D_ − *C*_0_).
(A16)

Solving for *E* yields the rotation-dependent energy levels *E_n,J_* as:

(A17)

where we note that the three parameters *C*_0_, *C*_1_ and *C*_2_ are functions of the rotational quantum number *J* as defined in Equation (A8).

For pure vibrational motion with *J* = 0, we have *C*_0_ = *C*_1_ = *C*_2_ = 0 and Equation (A17) reduces to the pure vibrational energy level:

(A18)

The dependence of *E*_*n,J*_ on the rotational quantum number *J* can be illustrated by taking Taylor series expansion of *E*_*n,J*_ with respect to the kinetic rotation energy *K* = ħ^2^*J*(*J* + 1)/(2*Mr*_0_^2^),

*E_n,J_* = *E*_*n*,0_ + *E_R_* + *E*_1*VR*_ + *E*_2*VR*_ + …,
(A19)

where the second term *E_R_* is attributed to a pure rotation motion:

(A20)

The third and fourth terms in *E_n,J_* are, respectively, the first-order and second-order vibration-rotation coupling energies:

(A21)

(A22)
